# POF (paclitaxel/oxaliplatin/5-fluorouracil/leucovorin) vs. SOX/CAPOX/FOLFOX as a postoperative adjuvant chemotherapy for curatively resected stage III gastric cancer: Study protocol for a randomized controlled trial, FNF-014 trial

**DOI:** 10.3389/fmed.2022.861777

**Published:** 2022-08-02

**Authors:** Liyu Su, Shen Zhao, Yi Yin, Feng Huang, Jinfeng Zhu, Luchuan Chen, FNF Independent Investigations Group, Rongbo Lin

**Affiliations:** ^1^Department of Gastrointestinal Medical Oncology, Fujian Cancer Hospital and Fujian Medical University Cancer Hospital, Fuzhou, China; ^2^Fujian Key Laboratory of Translational Cancer Medicine, Fuzhou, China; ^3^Department of Gastrointestinal Surgical Oncology, Fujian Cancer Hospital and Fujian Medical University Cancer Hospital, Fuzhou, China; ^4^Department of Medical Oncology, Quanzhou First Hospital, Quanzhou, China

**Keywords:** gastric cancer, adjuvant chemotherapy, POF, disease-free survival, survival

## Abstract

**Background:**

Postoperative chemotherapy is a standard treatment for stage II and III gastric cancer in Asia. With regard to single-agent or doublet, the need for improvement has consistently been pointed out because of the relatively poor outcome for patients with stage III gastric cancer. Triplet has shown significant survival benefits in the perioperative setting. We conducted a randomized, multicenter, phase III study to compare triplet to doublet regimens for patients with stage III gastric cancer.

**Methods:**

This is currently enrolling patients (*n* = 230) with pathologic stage III gastric cancer after D2 lymph node dissection and achieved R0 resection. Patients are randomized 1:1 and stratified by tumor stage (IIIA, IIIB, or IIIC, AJCC 8th) into POF or SOX/CAPOX/FOLFOX. S-1 and oxaliplatin (SOX): oxaliplatin 130 mg/m^2^ on day 1, oral S-1 80–120 mg/m^2^ divided by two on days 1–14 every 21 days for 8 cycles. Capecitabine and oxaliplatin (CAPOX): oxaliplatin 130 mg/m^2^ on day 1, oral capecitabine 1000 mg/m^2^ twice daily on days 1–14 every 21 days for 8 cycles. Folinic acid (or leucovorin), 5-fluorouracil and oxaliplatin (FOLFOX): oxaliplatin 85 mg/m^2^, levo-leucovorin 200 mg/m^2^, and 5-fluorouracil (5-FU) 400 mg/m^2^ bolus on day 1, then 5-FU 2400 mg/m^2^ continuous infusion over 46 h, every 14 days for 12 cycles. Three doublets were chosen by the clinicians. Paclitaxel, oxaliplatin, 5-fluorouracil, and leucovorin (POF): paclitaxel 135 mg/m^2^, followed by FOLFOX omitted 5-FU bolus, every 14 days for 12 cycles. The primary end point is 3-year disease-free survival (3-year-DFS). Secondary end points are overall survival (OS) and safety (any adverse event).

**Discussion:**

The results of this study will help establish postoperative clinical evidence for patients with locally advanced gastric adenocarcinoma or gastroesophageal junction adenocarcinoma.

**Clinical Trial Registration:**

[www.ClinicalTrials.gov], identifier [NCT0378826].

## Background

Gastric cancer (GC) is the fifth commonest cancer and the fourth leading cause of cancer mortality worldwide with over one million new cases in 2020 and an estimated 769,000 deaths (1). Most patients with gastric cancer are from China which led to a heavy health burden. The incident cases in China presented a similar trend, increasing from 317,340 cases in 1990 to 612,820 cases in 2019, and caused 421,540 deaths in 2019, corresponding to a 38% increase from the 305,470 deaths in 1990 (2).

Surgery is the optimal curative treatment for gastric cancer. Reports indicate that even after adequate gastric cancer resection including D2 lymphadenectomy, the prognosis of patients with advanced gastric cancer (AGC) remains dismal, which is mainly due to a high locoregional and distant failure (3). Therefore, it has come to be considered that stronger therapeutic effects with perioperative chemotherapy improve locoregional and distant control and survival. In Western civilizations, more patients are recommended to be given preoperative chemotherapy aiming at decreasing the tumor size and allowing for curative resection (4, 5). In contrast, the indication for neoadjuvant treatment is undefined in East Asia, where more patients are diagnosed with primarily resectable gastric cancer. For them, the standard of care is resection with a D2 lymphadenectomy followed by adjuvant chemotherapy (6).

The results of ACTS-GC demonstrated a sufficient effect of S-1 monotherapy for stage II and insufficient for stage III gastric cancer (7). After this, the feasibility of several combinations of anticancer drugs with S-1 was explored in the postoperative setting (8–10). JACCRO GC07 trial demonstrated the superiority of S-1 plus docetaxel to S-1 for 3-year relapse-free survival (3-year-RFS) (66 vs. 50%, *p* < 0.05) (11). Currently, CAPOX is recommended by many guidelines for postoperative therapy (12–15). Also further studies have demonstrated that the FOLFOX regimen is an effective and well-tolerated treatment for patients with gastric cancer (16). Regardless of single agent or doublet, the outcome of stage III gastric cancer patients is still very poor. Triplet [fluorouracil, leucovorin, oxaliplatin, and docetaxel (FLOT)] has shown significant survival benefits in perioperative setting with no related serious adverse events (17). Paclitaxel and FOLFOX (POF) regimens have proven to be more effective and tolerable than two-drug regimens (18, 19). We conducted a randomized, multicenter, phase III study to compare triplet to doublet regimens for patients with stage III gastric cancer. The main objective of this study is to prove the superiority of POF over SOX/CAPOX/FOLFOX in postoperative adjuvant setting for pathological stage III gastric adenocarcinoma (including adenocarcinoma of the gastroesophageal junction). Secondary objectives include 3-year overall survival, 5-year overall survival, 5-year disease-free survival, and adverse events.

## Methods/Design

### Study design

This is a multicentric, prospective, randomized, parallel-group, phase III clinical trial and will include patients from five tertiary hospitals in China. In December 2018, the study was registered with Clinical Trials Register (NCT03788226) under the name “A randomized phase III study comparing POF (paclitaxel/oxaliplatin/leucovorin/5-FU) with SOX/CAPOX/FOLFOX as a postoperative adjuvant chemotherapy for curatively resected stage III gastric cancer.” The trial protocol was reviewed and approved by the central ethics committee of the Fujian Cancer Hospital and local ethics committees of all participating hospitals and was conducted in accordance with the Helsinki Declaration and the good clinical practice guidelines. All participants provided written informed consent.

### Study population

The eligibility and exclusion criteria for the present prospective study were as follows:

Inclusion criteria

(1) Male or female aged 18–75 years.
(2) Primary histologically proven gastric adenocarcinoma (including adenocarcinoma of the gastroesophageal junction) of stage IIIA, IIIB, IIIC with no evidence of metastatic disease, R0 resection with D2 lymph node dissection with at least 15 lymph nodes were examined.
(3) Good performance status [Eastern Cooperative Oncology Group performance status score (ECOG PS) ≤ 1].
(4) No prior systemic anti-tumor treatment including chemotherapy, immunotherapy, or radiotherapy for gastric adenocarcinoma.
(5) Subjects must be able to take orally.
(6) No history of other malignant diseases in the past 5 years (except for cured skin cancer or cervical carcinoma *in situ*).
(7) No other concomitant medical conditions that required treatment.
(8) Initially treated with curative surgery followed by chemotherapy within 42 days.
(9) Adequate organ function based on the following parameters:(a) Absolute neutrophil count ≥ 1.5 × 10^3^/L, (b) Platelet count ≥ 100 × 10^9^/L, (c) Adequate renal function: creatinine ≤ 1.5 times the upper limit of normal (ULN) or glomerular filtration rate of creatinine clearance (calculated using the Cockcroft–Gault formula) > 50 ml/min, (d) Adequate hepatic function: alanine aminotransferase-to-aspartate aminotransferase ratio ≤ 2.5 × ULN or total bilirubin ≤ 1.5 × ULN.
(10) Life expectancy estimated to be more than 6 months.
(11) Patients who are willing and able to comply with the protocol during the study.
(12) All patients provided written informed consent prior to treatment.

Key exclusion criteria

(1) Patients with other primary malignancies, gastrointestinal bleeding, and definite contraindications for the use of corticosteroids.
(2) Uncontrolled active infection or serious concomitant systemic disorders.
(3) Patients known to be allergic or intolerant to the study drugs.
(4) Female patients during pregnancy or lactation, or subjects of childbearing age who refuse contraception.
(5) Patients who were not suitable for the enrollment in this study judged by the investigator.

### Recruitment

**FIGURE 1:**
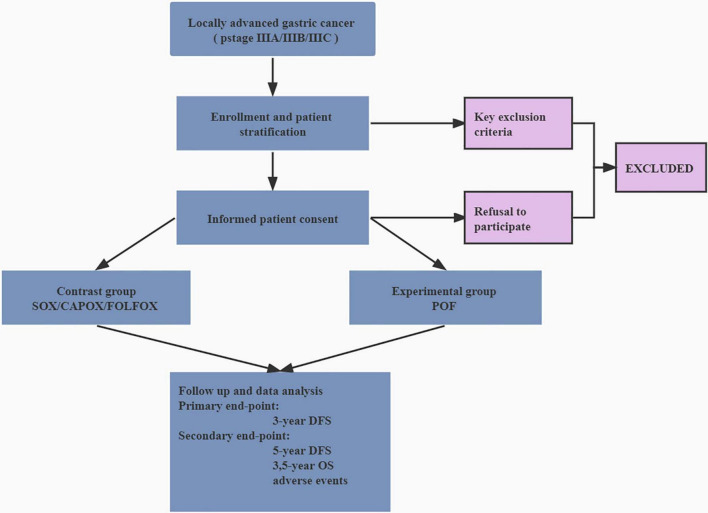
Flow diagram of the trial. DFS, disease-free survival; OS, overall survival; SOX, oxaliplatin and S-1; CAPOX, oxaliplatin and capecitabine; FOLFOX, folinic acid (or leucovorin), 5-fluorouracil and oxaliplatin; POF, paclitaxel, folinic acid (or leucovorin), 5-fluorouracil, and oxaliplatin.

Study candidates will be identified by a study physician, who will explain the study to candidates or authorized surrogates. Written informed consent will be obtained from candidates or authorized surrogates prior to inclusion in the study. A flow diagram is shown in Figure 1, and the SPIRIT figure is shown in Table 1.

**TABLE 1:** SPIRIT figure showing time points for enrollment, interventions, and assessment.

Tests	Study period				
	Enrollment	Allocation	Treatment period*^a^*	Follow-up period*^b^*	Closeout
**Assessment of eligibility**					
Informed consent	X				
Inclusion and exclusion criteria	X				
Demographic data	X				
Clinical history	X				
Allocation		X			
**Interventions**					
Intervention A			X		
Intervention B			X		
**Assessment of efficacy**					
Tumor markers	X	X	X	X	X
Imaging results	X	X	X	X	X
**Assessment of safety**					
Physical examination	X	X	X	X	X
Blood chemistry (whole blood count, liver-renal function test, etc.)	X	X	X	X	X
ECG examination	X	X	X	X	X
Assessment of adverse events			X	X	X
**Other assessments**					
Nutritional status assessment	X	X	X	X	X
Other treatments	X	X	X	X	X
Compliance	X	X	X	X	X
Survival	X	X	X	X	X

ECG, electrocardiography.

^a^Patients are followed up every 3 months of treatment.

^b^Patients are to visit the clinic every 3 months for 2 years after surgery and 6 months thereafter.

### Randomization

Before the start of the study, a computer-generated sequence of random numbers was placed in a series of plain, sealed envelopes with patient numbers on them by the research nurse. These envelopes were created and kept at the School of Public Health of Fujian Medical University and only opened at the time of subject randomization, again by the research nurse. Individuals directly involved in the study had no access to these envelopes. Patients will be assigned to one of the two therapy groups on a 1:1 ratio. Factors such as pathological stage (IIIA, IIIB, and IIIC) will be considered when allocating the subjects into one of the two groups. The statistician and research nurse are blinded to the recruitment procedure prior to initiation of the trial. Because this was an open-label trial, the patients and physicians were not masked to the study groups. A site radiologist who assessed tumor radiographic responses, a central radiologist who verified it, and a statistician who statistically analyzed the data, were blinded to the study groups.

### Intervention

**FIGURE 2:**
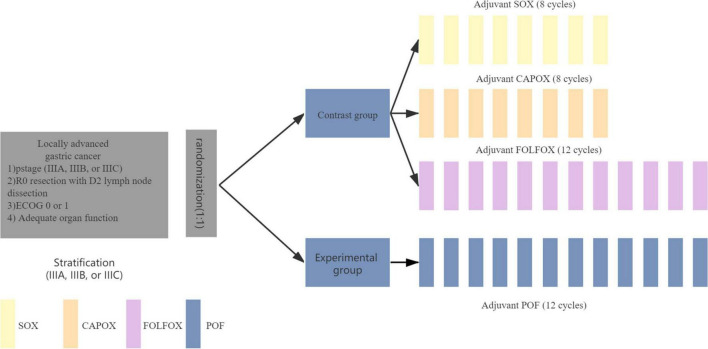
Study treatment. SOX, oxaliplatin and S-1; CAPOX, oxaliplatin and capecitabine; FOLFOX, folinic acid (or leucovorin), 5-fluorouracil and oxaliplatin; POF, paclitaxel, folinic acid (or leucovorin), 5-fluorouracil, and oxaliplatin.

Patients are randomized 1:1 and stratified by tumor stage (IIIA, IIIB, or IIIC, AJCC 8th) into POF or SOX/CAPOX/FOLFOX (chosen by the clinicians) (Figure 2). The allocated treatment was initiated within 2 weeks of randomization and 4–8 weeks after surgery. SOX: oxaliplatin 130 mg/m^2^ on day 1, oral S-1 on days 1–14 every 21 days for 8 cycles which dose dependent on body surface area (body surface area < 1.25 m^2^, 40 mg twice a day; body surface area 1.25–1.5 m^2^, 50 mg twice a day; and body surface area > 1.5 m^2^, 60 mg twice a day). CAPOX: oxaliplatin 130 mg/m^2^ on day 1, oral capecitabine 1000 mg/m^2^ twice daily on days 1–14 every 21 days for 8 cycles. FOLFOX: oxaliplatin 85 mg/m^2^, levo-leucovorin 200 mg/m^2^, and 5-FU 400 mg/m^2^ bolus on day 1, then 5-FU 2400 mg/m^2^ continuous infusion over 46 h, every 14 days for 12 cycles. Three doublets were chosen by the clinicians. POF: paclitaxel 135 mg/m^2^, followed by FOLFOX omitted 5-FU bolus, every 14 days for 12 cycles. Antiemetic prophylaxis was given according to local protocols; granulocyte colony-stimulating factor was not recommended as primary prophylaxis. Pre-medications (antihistamine, corticosteroid, and H2 receptor antagonist) were administrated for prophylaxis of hypersensitivity reactions to paclitaxel. Doses were modified in response to toxicities (Supplementary Material).

### Study endpoints

The primary end point is 3-year disease-free survival. The time was calculated from the date of randomization to the date of recurrence of the original gastric cancer, development of a new gastric cancer, or the date of the death from any cause. Secondary end points are 3-year overall survival, 5-year overall survival, 5-year disease-free survival, and adverse events. OS is defined as the time from the initial diagnosis to death from any cause.

### Follow-up

Patients are to visit the clinic every 3 months for 2 years after surgery and 6 months thereafter. Follow-up appointments will include physical examination, complete blood count, routine chemistry including liver and kidney function tests, and serum carcinoembryonic antigen test. Chest radiography and abdominal and pelvic computed tomography will be performed every 6 months for 5 years. A routine gastroscope will also be performed once a year after surgery.

### Evaluation of safety

Adverse events will be evaluated according to CTCAE version 5.0. The occurrence of adverse events from enrollment to 1 year after treatment will be recorded for all patients. Severe adverse events during hospital stay and after discharge will be reported to the study coordinator and promotor. If severe adverse events occur, follow-up will be performed until remission or until a diagnosis is made and its association with the study medication is established.

### Sample size

The primary purpose of this study is to test the superiority of triplet regimen (POF) to doublet (SOX/CAPOX/FOLFOX) adjuvant chemotherapy for curatively resected stage III gastric cancer. This study is based on the results of the RESOLVE trial, which showed a 3-year DFS of 55% in gastric cancer with stage III after the operation. We estimated that the experimental group (arm POF) will have a 3-year DFS of 67% and a superiority margin of 12% (20). With a statistical power of 80%, a two-sided α error of 5%, and a 10% dropout rate, we estimated a total of 230 patients for this study, with 115 patients for each arm.

### Statistics

Our analysis will be based on an intention-to-treat population and a per-protocol population. Continuous and categorical variables will be analyzed using the Mann–Whitney test and Fisher’s exact test, respectively. Time-to-event curves for disease-free survival and overall survival were calculated with the Kaplan-Meier method and compared by the log-rank test. An accurate 95% confidence interval of the overall survival and safety analysis will be estimated. The significance level is set at *p* < 0.05, and all statistical tests will be two-sided. The risk ratio will be estimated using a stratified Cox regression model. Data were analyzed using SPSS version 24.0 (IBM Corp., Armonk, NY, United States).

When half of the patients (*n* = 115) were enrolled and 32 weeks after the last patient enrolled, a planned interim analysis of the safety data was performed.

### Data processing and auditing

Data from each patient will be collected and anonymized by a study member in a data log. Each study center will send their data logs to the coordinating center. The principal investigator will enter data from data logs into a single database for all study patients. An interim analysis will be performed by an independent data monitoring committee. The study will be interrupted only if the severe adverse events are significantly higher in the three drugs group (POF) as compared with the two drugs group (SOX/CAPOX/FOLFOX). The final analysis will be carried out when the sample size has been reached. The coordinating center will be responsible for auditing the trial independently from investigators yearly.

## Discussion

China is a country with a high incidence of gastric cancer, and the burden of disease is serious. Surgical resection is the only possible cure for patients with gastric cancer, and the prognosis of gastric cancer is closely tied to the tumor stage and treatment. In China, more patients are diagnosed with primarily resectable gastric cancer. For them, adjuvant therapy is a standard with no regimen selection criteria. This study aims to compare the effect of adjuvant chemotherapy with POF (paclitaxel/oxaliplatin/5-fluorouracil/leucovorin) vs. SOX/CAPOX/FOLFOX for gastric cancer after D2 gastrectomy, to explore which treatment is better. The ACTS-GC study and the CLASSIC study confirmed the efficacy of 1 year S-1 and 6 months CAPOX in adjuvant chemotherapy, respectively (7, 21). Compared with surgery alone, the single agent or doublet increases the RFS rate or DFS rate in the value of 10–15%, and the value of 3-year OS rate is 5–10%. However, the efficacy of S-1 monotherapy or CAPOX remains inadequate in stage III patients. Recent studies have demonstrated that SOX was non-inferior to CAPOX in patients with locally AGC who had D2 gastrectomy (22). 3-year DFS was 51.1% in the CAPOX group and 56.5% in the SOX group. The most frequent grade 3–4 adverse event was neutropenia (12% in the CAPOX group, 8% in the SOX group). No treatment-related deaths were reported. The above-mentioned results lead to the conclusion that single agent or doublet may improve the survival of AGC patients with D2 gastrectomy. However, even when administered with doublet treatment strategies, the prognosis of patients with locally AGC who had D2 gastrectomy remains dismal. Thus, it has come to be considered that stronger therapeutic effects with postoperative adjuvant chemotherapy should be sought. Triplet has shown substantial survival benefits in the perioperative environment. From the FLOT4 trial, perioperative FLOT improved overall survival compared with perioperative ECF/ECX (17). Modified docetaxel, cisplatin, and 5-FU (mDCF) and paclitaxel plus FOLFOX (POF) regimens were found to be more efficient and tolerable than two-drug regimens in randomized trials (18, 23). This study was performed to compare triplet and doublet diets in patients with stage III gastric cancer. And we’re running this trial for patients with stage III disease, expect to expand and optimize the existing therapies.

Together, the results of this study will provide prospective multicenter data and contribute to available adjuvant treatment data for postoperative stage III gastric cancer.

## Ethics statement

The studies involving human participants were reviewed and approved by Fujian Cancer Hospital Ethics Committees. The patients/participants provided their written informed consent to participate in this study.

## Author contributions

RL conceived and designed the study and was responsible for the final decision and submission. SZ and LS provided administrative, technical, and material support. YY and FH were responsible for data cleaning and analysis. LS wrote the manuscript. JZ and LC polished and revised the manuscript. RL and LS reviewed and finalized the manuscript. RL, SZ, and LS were responsible for the collection and sorting of the data. All authors participated in revising it critically and approved the final version to be submitted.

## Conflict of interest

The authors declare that the research was conducted in the absence of any commercial or financial relationships that could be construed as a potential conflict of interest.

## Publisher’s note

All claims expressed in this article are solely those of the authors and do not necessarily represent those of their affiliated organizations, or those of the publisher, the editors and the reviewers. Any product that may be evaluated in this article, or claim that may be made by its manufacturer, is not guaranteed or endorsed by the publisher.
