# Supplementary material for: POF (paclitaxel/oxaliplatin/5-fluorouracil/leucovorin) vs. SOX/CAPOX/FOLFOX as a postoperative adjuvant chemotherapy for curatively resected stage III gastric cancer: Study protocol for a randomized controlled trial, FNF-014 trial
Source: Front Med (Lausanne). 2022 Aug 2;9:861777. doi: 10.3389/fmed.2022.861777 (PMC9380469; doi:10.3389/fmed.2022.861777)
Supplement: Supplementary file 1 [file Data_Sheet_1.docx]

Supplementary Material

**Treatment modification**

To ensure standardization across study sites and treatment regimens, dose delay should be carried out before dose modification as detailed in Tables 1 and 2.

**Dose delay**

For a grade 3 or 4 hematological or non-hematological AE, treatment delay should be implemented according to Table 1. The duration of treatment delay in any cycle shall not exceed 14 days to insure adequate drug dose intensity. If a delay exceeds 14 days, the participant is discontinued from protocol therapy, although tumor evaluation will still be carried out.

**Dose modification**

Guidelines for dose modification are provided in Table 2. In principle, dose adjustments should be carried out according to the AE spectrum of the drug, and specific modifications or delays should be according to the judgment of the investigator and best clinical practice. If a dose is reduced, it is not to be returned to the previous level.

**
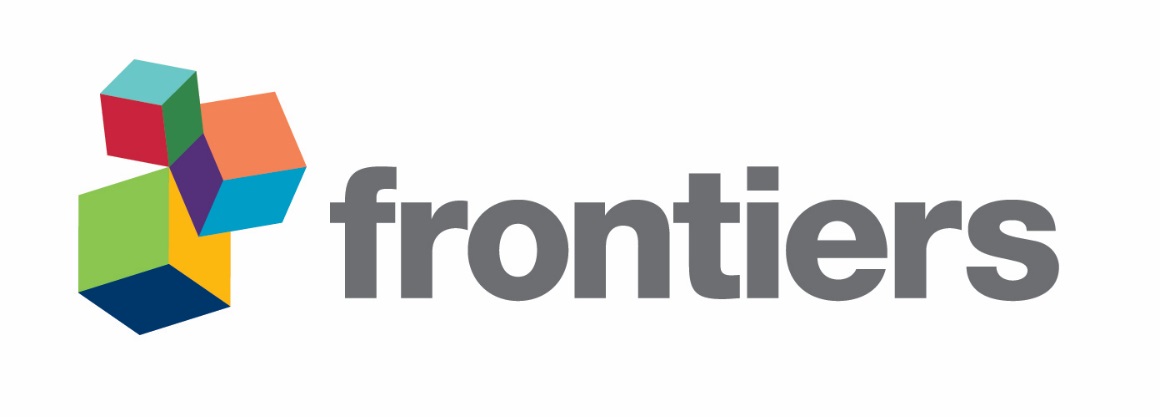
**

**Table 1. Treatment modification**

* Grading according to NCI-CTC AE 4.03

| Toxicity | Paclitaxel | Oxaliplatin | Fluorouracil |
| --- | --- | --- | --- |
| Grade 3 Hematological toxicity (non-leukopenia) | Hold until NCI CTCAE ≤grade 1; resume at same dose. If NCI CTCAE ≥grade 3 again, resume at reduced dose. | Hold until NCI CTCAE ≤grade 1; resume at same dose. If NCI CTCAE ≥grade 3 again, resume at reduced dose. | Hold until NCI CTCAE ≤grade 1, resume at same dose. If NCI CTCAE ≥grade 3 again, resume at reduced dose. |
| Grade 4 Hematological toxicity (non-leukopenia) | Hold until NCI CTCAE ≤grade 1; resume at reduced dose. | Hold until NCI CTCAE ≤grade 1; resume at reduced dose. | Hold until NCI CTCAE ≤grade 1, resume at reduced dose. |
| Grade 3 or 4 Leukopenia | Hold until NCI CTCAE ≤grade 2; dose reduction at investigator’s discretion. | Hold until NCI CTCAE ≤grade 2; dose reduction at investigator’s discretion. | Hold until NCI CTCAE ≤grade 2; dose reduction at investigator’s discretion. |
| Grade 3 Neurotoxicity | Permanently discontinue | Permanently discontinue | Permanently discontinue |
| Grade 3 Diarrhea, Stomatitis,  Hand/foot syndrome | Hold until NCI CTCAE ≤grade 1; resume at same dose. | Hold until NCI CTCAE ≤grade 1; resume at same dose. | Hold until NCI CTCAE ≤grade 1; resume at same dose. If NCI CTCAE ≥grade 3 again, resume at reduced dose. |
| Grade 4 Diarrhea, Stomatitis,  Hand/foot syndrome | Hold until NCI CTCAE ≤grade 1; resume at same dose. | Hold until NCI CTCAE ≤grade 1; resume at same dose. | Hold until NCI CTCAE ≤grade 1; resume at reduced dose. |
| Grade 3  Allergy | Investigator decision whether to continue. | Investigator decision whether to continue. | Investigator decision whether to continue. |
| Grade 4  Allergy | Permanently discontinue | Permanently discontinue | Permanently discontinue |
| Other drug- related  Grade 3 or 4 toxicities | Hold until NCI CTCAE ≤grade 1 (for elevated transaminase with liver metastases, hold until NCI CTCAE ≤grade 2); resume at same dose. If NCI CTCAE ≥grade 3 again, resume at reduced dose. | Hold until NCI CTCAE ≤grade 1 (for elevated transaminase with liver metastases, hold until NCI CTCAE ≤grade 2); resume at same dose. If NCI CTCAE ≥grade 3 again, resume at reduced dose. | Hold until NCI CTCAE ≤grade 1 (for elevated transaminase with liver metastases, hold until NCI CTCAE ≤grade 2); resume at same dose. If NCI CTCAE ≥grade 3 again, resume at reduced dose. |

**Table 2. Dose reduction**

| Drug | Initial dose level | First dose reduction | Second dose reduction |
| --- | --- | --- | --- |
| IV Paclitaxel  Oxaliplatin  Fluorouracil | 135 mg/m^2^  85 mg/m^2^  2400 mg/m^2^ | 120 mg/m^2^  75 mg/m^2^  2000 mg/m^2^ | No further reduction; delay treatment until toxicity resolves |
